# Characterization of a novel adult murine immortalized microglial cell line and its activation by amyloid-beta

**DOI:** 10.1186/s12974-016-0484-z

**Published:** 2016-01-27

**Authors:** Ryan C. McCarthy, Dah-Yuu Lu, Ahmed Alkhateeb, Andrew M. Gardeck, Chih-Hao Lee, Marianne Wessling-Resnick

**Affiliations:** Department of Genetics and Complex Diseases, Harvard School of Public Health, 655 Huntington Avenue, Boston, MA 02115 USA; Present Address: Graduate Institute of Neural and Cognitive Sciences, China Medical University, Taichung, Taiwan, Republic of China; Present Address: The Wellman Center for Photomedicine, Massachusetts General Hospital and Harvard Medical School, Boston, MA USA

**Keywords:** BV-2 cells, Neurodegeneration, Microglia, Neuroinflammation, IMG cells, Amyloid-beta

## Abstract

**Background:**

Alzheimer’s disease is associated with amyloid-beta (Aβ)-induced microglia activation. This pro-inflammatory response promotes neuronal damage, and therapies are sought to limit microglial activation. Screening efforts to develop new pharmacological inhibitors require a robust in vitro cell system. Current models lack significant responses to Aβ, and their use in examining age-related neurodegenerative diseases is questionable. For example, the commonly used BV-2 microglial line was derived from embryonic mononuclear cells and its activation by various stimuli is limited. To this end, we have established a new immortalized microglial (IMG) cell line from adult murine brain. The objective of this study was to characterize Aβ-induced activation of IMG cells, and here, we demonstrate the ability of cannabinoids to significantly reduce this inflammatory response.

**Methods:**

Microglial cells derived from adult murine brain were immortalized via infection with the v-raf/v-myc retrovirus under conditions that selectively promote microglia growth. The presence or absence of markers CD11b and F4/80 (microglial), NeuN (neuronal), and GFAP (astrocytic) was assessed by immunofluorescence microscopy and western blotting. Using IMG and BV-2 cells, levels of pro- and anti-inflammatory transcripts in response to extracellular stimuli were determined by quantitative PCR (qPCR). Phagocytosis of fluorescent beads and fluorescein isothiocyanate (FITC)-labeled Aβ oligomers was assessed using flow cytometry and fluorescence microscopy. FITC-Aβ uptake was quantified using a fluorescence plate reader. The ability of cannabinoids to mitigate Aβ-induced expression of inducible nitric oxide synthase (iNOS) was evaluated.

**Results:**

IMG cells express the microglial markers CD11b and F4/80 but not NeuN or GFAP. Relative to BV-2 cells, IMG cells increased iNOS (>200-fold) and Arg-1 (>100-fold) in response to pro- and anti-inflammatory stimuli. IMG cells phagocytose foreign particles and Aβ oligomers, with the latter trafficked to phagolysosomes. Aβ-induced activation of IMG cells was suppressed by delta-9-tetrahydrocannabinol and the CB2-selective agonist JWH-015 in a time- and concentration-dependent manner.

**Conclusions:**

IMG cells recapitulate key features of microglial cell activation. As an example of their potential pharmacological use, cannabinoids were shown to reduce activation of Aβ-induced iNOS gene expression. IMG cells hold promising potential for drug screening, mechanistic studies, and functional investigations directed towards understanding how Aβ interacts with microglia.

**Electronic supplementary material:**

The online version of this article (doi:10.1186/s12974-016-0484-z) contains supplementary material, which is available to authorized users.

## Background

Microglial cells, often thought of as the resident macrophages of the central nervous system (CNS), act as the immune cells of the brain and spinal cord. Microglia become activated by disturbances in the homeostasis of their local microenvironment. Activation of microglial cells results in a cascade of phenotypic changes including, but not limited to, morphology, transcription, and cytokine production. While there are varying degrees of microglia activation [1], two main polarized states are established: a pro-inflammatory reactive state induced by exposure to stimuli like lipopolysaccharide (LPS) and interferon-γ (IFNγ) and an anti-inflammatory state to promote repair and resolution of inflammation, which is induced by factors such as interleukin-4 (IL-4) and interleukin-13 (IL-13) [2, 3].

The ability to polarize between reactive and repair states allows microglia to actively transition from an immune-stimulating antimicrobial phenotype to one that supports tissue repair and resolution of inflammation [1]. Dysregulation of the activation state of microglial cells can be detrimental to CNS health. Several neurodegenerative diseases including Alzheimer’s disease and Parkinson’s disease have been attributed to chronically activated pro-inflammatory microglial cells [4–10]. Chronic activation of microglial cells is thought to result from multiple stimuli ranging from systemic infection, misfolded proteins, or cellular debris within the CNS [5]. In the case of the Alzheimer’s disease brain, microglia are activated by amyloid-β (Aβ) peptides, which are cleared from the interstitium by phagocytosis. As the disease persists, microglia become chronically activated by Aβ peptides and produce excessive amounts of pro-inflammatory cytokines leading to autocrine reduction of microglial Aβ receptors and ultimately decreased Aβ clearance from the interstitium [11].

Regardless of the provoking stimuli, efforts to target activated microglia for the treatment of certain neurodegenerative diseases include pharmacological re-polarization to the more anti-inflammatory phenotype [2, 12]; cannabinoids represent one class of pharmacological agents currently being examined. Unfortunately, studies of microglial cell function are limited by low yields of primary microglial cells (approx. 500,000 cells per adult mouse brain) along with potential activation during isolation. Several immortalized microglia cell lines have been generated [13, 14] and provide experimental advantages of a homogeneous population of cells that can proliferate more rapidly. Most microglial cell studies have relied on the murine BV-2 cell line which was immortalized by infection of embryonic brain mononuclear cells with a v-raf/v-myc oncogene-carrying retrovirus [14, 15]. However, more recent evidence suggests that microglial cells of the adult brain are derived from myeloid progenitors [16]. Therefore, the embryonic origin of BV-2 cells raises questions about their epigenetic state and whether they truly resemble resident microglia in the adult brain.

Here, we report the generation and characterization of a novel cell line using microglia purified from the adult mouse brain. These cells were established using the Percoll gradient isolation followed by culture conditions that selectively promote microglial cell growth [17, 18]. This study characterizes the properties of the immortalized microglia (IMG cells), which express markers specific to primary adult microglial cells (CD11b and F4/80). IMG cells respond to pro-inflammatory (LPS and Aβ) or anti-inflammatory (IL-4) stimuli. The changes induced by LPS, Aβ, and IL-4 are far greater than those induced in BV-2 cells. IMG cells provide a model system for drug candidate screening as evidenced by inhibition of Aβ-induced M1 activation by the cannabinoids delta-9-tetrahydrocannabinol (THC) and JWH-015. Lastly, we demonstrate that IMG cells retain the ability to engulf fluorescent beads and fluorescein isothiocyanate (FITC)-labeled Aβ by phagocytosis, functions that are important to explore experimentally to further our understanding of complex Aβ-microglia interactions in Alzheimer’s disease.

## Methods

### Cell culture and reagents

IMG, C6 glioma, and BV-2 cells were cultured in Dulbecco’s modified Eagle medium (DMEM) with high glucose (4.5 g/L), 10 % fetal bovine serum (FBS) and penicillin/streptomycin (100 U/mL). SH-SY5Y cells were maintained in DMEM/F12 (1:1 ratio) media with 10 % FBS and penicillin/streptomycin (100 U/mL). IFNγ, IL-1β, TNF-α, IL-4, IL-6, and IL-13 were purchased from Peprotech (Rocky Hill, NJ). LPS, Ac-YVAD-CMK, delta-9-tetrahydrocannabinol, and JWH-015 were purchased from Sigma Aldrich. Adenosine triphosphate (ATP) was purchased from Amersham Biosciences.

### Primary microglia isolation and generation of IMG cell line

Microglia were purified from adult brain using gradient isolation methods [19]. Proliferation and retroviral infection was carried out in medium conditioned with growth factors GM-CSF and M-CSF to selectively support microglial growth. Briefly, 8-week-old C57BL/6J mice were perfused with ice-cold phosphate-buffered saline (PBS) through the left ventricle. After collagenase digestion of brain slices, and debris removal, microglia were isolated on Percoll gradients (30 %–37 %–70 %) and collected at the 37–70 % interface. Microglial cells were maintained in DMEM (5 mM glucose), 10 % FBS, and 1 % P/S using 30 % L929 cell-conditioned medium to induce proliferation of microglial cells. Cells proliferated at days 5–10 after plating. To immortalize the cells, they were re-plated and infected with v-raf/v-myc retrovirus (J2-conditioned medium [20]), supplemented with 30 % L929-conditioned media and 4 μg/mL polybrene. The next day, the medium was replaced by DMEM (4.5 g/L glucose), 10 % FBS, and 1 % P/S.

### Flow cytometry

IMG cells were grown on a 10-cm tissue culture dish until 80 % confluent. After addition of 5 mL fresh media, cells were lifted off the dish using a cell scraper. IMG cells were resuspended to 1 × 10^8^ cells/mL and incubated with fluorescently conjugated CD11b (Alexa 647 conjugate; Serotec), F4/80 (allophycocyanin (APC) conjugate; Caltag), or appropriate isotype control (BioLegend) antibodies (1:10 dilution) in the dark for 15 min at 4 °C. Cells were then washed three times with 2 mL cell-staining buffer (BioLegend). After washing, cells were resuspended in cell-staining buffer and were analyzed by flow cytometry (FACSCalibur, BD Biosciences). Acquired data were analyzed using FlowJo data analysis software (FlowJo, LLC).

### Fluorescence microscopy

IMG and SH-SY5Y cells grown on poly-d-lysine-coated coverslips were fixed for 20 min with 4 % formaldehyde at 4 °C in PBS containing 0.5 mM MgCl_2_ and 1 mM CaCl_2_ (PBS^++^). Cells were then permeabilized with 0.5 % Triton-X100 in PBS^++^ for 5 min at room temperature. After blocking with 1 % bovine serum albumin (BSA) and 0.3 M glycine in PBS^++^ for 1 h at room temperature, cells were incubated for 1 h at room temperature with anti-NeuN (Millipore, MAB377) (1:100), anti-F4/80 (Caltag, MF48020) (1:100), or anti-CD11b (Serotec) (1:100). Cells were then washed and incubated for 1 h at room temperature with Alexa Fluor 568-conjugated anti-rabbit or anti-mouse antibody (1:1000, Invitrogen) in 1 % BSA in PBS^++^. Coverslips were mounted onto glass slides using DakoCytomation fluorescent mounting medium (Carpinteria, CA). Images were obtained using a Zeiss AxioImager Z1 Axiophot wide-field fluorescence microscope and were analyzed by Zeiss AxioVision software (Zeiss, Thornwood, NY).

### Immunoblot

IMG and C6 glioma cells were incubated for 24 h in full growth medium plus 100 ng/mL IL-6 to allow for astrocytic differentiation of C6 glioma cells [21]. For cytosolic protein extracts, cells were lysed in hypotonic buffer using 1 % NP-40 plus protease inhibitors (Calbiochem Cat. No. 539134; 1:100 dilution) on ice followed by centrifugation to separate the remaining nuclei (pellet) from the cytosolic fraction (supernatant). The nuclei-containing pellets were lysed with RIPA buffer plus protease inhibitors for 30 min on ice. Protein concentration was determined, and 30–50 μg of protein/sample was heated for 5 min at 95 °C, cooled on ice, and then resolved on a 4–15 % SDS-PAGE gel (10 % SDS-PAGE gel used for cytosolic and nuclear extracts). The protein was transferred onto a nitrocellulose membrane (0.2 μm) using a Trans-blot turbo transfer system (Bio-Rad, Hercules, CA). The resulting membrane was blocked for 1 h at room temperature in TBST (Tris-buffered saline plus 0.05 % Tween-20) plus 5 % milk. After three washes with TBST, the membrane was incubated with primary mouse monoclonal GFAP (GA5) antibody (1:1000 dilution; Cell Signaling Technology, Beverly, MA), rabbit monoclonal NeuN antibody (1:1000 dilution; Abcam Inc., Cambridge, MA), rat monoclonal F4/80 antibody (1:10,000 dilution; AbD Serotec), rabbit polyclonal β-tubulin antibody (1:500; Abcam), or rabbit monoclonal Lamin B1 antibody (1:10,000 dilution; Abcam) in TBST plus 5 % milk overnight at 4 °C. The membrane was washed three times with TBST and then was incubated for 1 h at room temperature with IRDye 800CW donkey anti-mouse or anti-rabbit IgG (1:5000 dilution; Li-Cor, Lincoln, NE) in TBST 5 % milk (anti-rat HRP 1:5000 dilution was used to probe for F4/80 primary antibody). The membrane was washed three times with TBST and was imaged using Li-Cor Odyssey 2.1 infrared detection technology.

### Quantitative RT-PCR

Total RNA was extracted from IMG or BV-2 cells using TRIzol reagent (Invitrogen, Carlsbad, CA) as per the manufacturer’s instructions. For analysis of adult microglial markers, microglia were pre-incubated for 5 days in full growth medium plus mouse recombinant carrier-free MCSF (10 ng/mL; R&D Systems) and recombinant TGFβ1 (50 ng/mL; Miltenyi Biotec) as described [26]. RNA was purified and on-column DNAse treated using the Direct-zol RNA Miniprep Kit from Zymo-research (Irvine, CA) as per the manufacturer’s instructions. Purified RNA was then reverse-transcribed using the SuperScript® III First-Strand Synthesis System (Invitrogen) along with oligo(dT)20 primers and random hexamers. Quantitative PCR was performed using iTaq Universal SYBR green Supermix (Bio-Rad, Hercules, CA) and the StepOnePlus Real-Time PCR System (Life Technologies, Grand Island, NY). In all cases, 36B4 was used as an internal control. Primers used for quantitative PCR (qPCR) are listed in Table 1.

**Table 1:** Primer list used for qPCR

Transcript	Forward primer	Reverse primer
Mouse 36B4	AGATGCAGCAGATCCGCAT	GTTCTTGCCCATCAGCACC
Mouse iNOS	GTTCTCAGCCCAACAATACAAGA	GTGGACGGGTCGATGTCAC
Mouse TNF-α	AAATGGCCTCCCTCTCATCAG	GTCACTCGAATTTTGAGAAGATGATC
Mouse IL-1β	AGCTTCAGGCAGGCAGTATC	AAGGTCCACGGGAAAGACAC
Mouse Arg-1	CACAGTCTGGCAGTTGGAAG	GGGAGTGTTGATGTCAGTGTG
Mouse Mgl1	GCGAAGGCTTACAATGATATACGAAAACCTCC	GCGCTCAGACGAGAGCTCCTAGCTCTCC
Mouse Ym1	AGAAGGGAGTTTCAAACCTGGT	GTCTTGCTCATGTGTGTAAGTGA
Mouse Itgb5	CTCCAGGGCCCGTTATGAAA	AGGCGAAATCGACAGTGTGT
Mouse Tgfb1	AGGGCTACCATGCCAACTTC	CCACGTAGTAGACGATGGGC
Mouse Fcrls	AAGTGCGTTTGGTGAATGGC	CACAGCCACATCCCTCATGT
Mouse Sall1	TGCTGACCAACGACTCTTCC	ACTGGGGTGGGAGATAGACC

### Enzyme-linked immunosorbent assays

Mini enzyme-linked immunosorbent assay (ELISA) development kits (Peprotech, Rocky Hill, NJ) were used to detect murine TNF-α, IL-6, and IL-1β expression by IMG cells. Buffers used throughout this protocol were purchased as an ELISA Buffer Kit from Peprotech (Catalog #900-K00). Briefly, IMG cells were incubated for 16 h with either LPS (10 ng/mL) or IL-4 (10 ng/mL) in a six-well tissue culture dish. Alternatively, IMG cells were incubated for 6 h with or without LPS (10 ng/mL) in 1 mL of full growth media at 37 °C 5 % CO_2_. Ac-YVAD-CMK (40 μM) was then added to the appropriate wells for 5 min prior to the addition of ATP (5 mM) for 30 min. After the 6- or 16-h incubation, the conditioned media were collected and the cells were washed three times with PBS and were lysed for 30 min at 4 °C with 1 % NP-40 plus protease inhibitors. Appropriate capture antibody was adhered to wells of a 96-well plate overnight at room temperature. After repeated washes, the wells were blocked with 1 % BSA in PBS for 1 h at room temperature followed by multiple washes. For each condition, 100-μL aliquots of cell lysates were added to each well in triplicate for 2 h at room temperature. The plate was washed repeatedly, and detection antibody (0.5 μg/mL) was added and incubated for 1 h at room temperature. Plates were washed and incubated with avidin-HRP conjugate (1:2000 dilution) for 30 min at room temperature. Lastly, the plates were washed, and 2,2′-Azinobis [3-ethylbenzothiazoline-6-sulfonic acid]-diammonium salt (ABTS) liquid substrate was added to each well. Color development (405 nm) was monitored using a BioTek Synergy 2 spectrophotometer (Winooski, VT).

### IL-1β immunoblot

IMG cells in a six-well poly-d-lysine-coated tissue culture dish were treated for 6 h with or without LPS (10 ng/mL) in 1 mL of full growth media at 37 °C 5 % CO_2_. Ac-YVAD-CMK (40 μM) was then added to the appropriate wells for 5 min prior to the addition of ATP (5 mM) for 30 min. The IMG cell-conditioned media were then collected and concentrated ten times from 1 mL to 100 μL using a 10K MWCO Nanosep Omega centrifugal device (PALL, Ann Arbor, MI). Seven microliters of this media was heated at 95 °C for 5 min and then resolved on a 4–20 % SDS-PAGE gel. The protein was then transferred onto a 0.2-μm nitrocellulose membrane using a wet-transfer apparatus at 100 V for 60 min. The membrane was blocked with 5 % milk in TBST at 4 °C for 1 h prior to incubation overnight at 4 °C with goat polyclonal IL-1β antibody (1:500 dilution; Santa Cruz Biotechnology, Inc.). The membrane was washed three times with TBST and then was incubated for 1 h at room temperature with IRDye 800CW donkey anti-goat IgG (1:5000 dilution; Li-Cor) in TBST 3 % milk. The membrane was washed three times with TBST and was imaged using Li-Cor Odyssey 2.1 infrared detection technology.

### Phagocytosis assays

IMG cells were seeded into two six-well tissue culture plates (0.5 × 10^6^ cells/well) and allowed to adhere for 2 h, after which time the media were exchanged with fresh media to remove non-adherent cells. IMG cells were allowed to grow for 16 h at 37 °C/5 % CO_2_ prior to the start of the assay. Sixty-five-microliter aliquots of carboxylate-modified polystyrene fluorescent yellow-green latex beads (YG beads) (Sigma Aldrich, Cat# L4655) were diluted in 6.5-mL aliquots of pre-warmed (37 °C) or pre-chilled (4 °C) growth media. Media were removed from IMG cells, and 1 mL of YG bead-containing media was added to each well. IMG cells were immediately incubated at 37 °C or chilled at 4 °C for 1 h. The remaining steps were strictly performed on ice. To remove non-internalized beads, cells were washed five times with 2 mL/well ice-cold PBS. After washing, the IMG cells were incubated with 2 mL/well ice-cold PBS containing 2 mM EDTA for 10 min at 4 °C. Cells were removed from the dish by titration and transferred to 15-mL conical tubes. Cells were collected by centrifugation at 300×*g* for 6 min at 4 °C. Cell pellets were resuspended in PBS containing 2 mM EDTA. IMG cell-acquired YG beads were quantified by flow cytometry, and data were analyzed.

### Amyloid-beta assays

Amyloid-beta (1–42), FITC-amyloid-beta (1–42), and scrambled amyloid-beta (1–42) were purchased from rPeptide (Bogart, GA). Briefly, HFIP-prepared peptide was resuspended with DMSO (0.1 mg in 10 μL) and then diluted 1:10 with Ham’s F-12 nutrient mix and incubated for 24 h at 4 °C as described [22, 23]. Both oligomeric and fibrillar Aβ_1–42_ were detected by dot blot analyses using species-specific antibodies (Additional file 1: Figure S1). IMG phagocytosis of FITC-Aβ was performed using cells seeded into a 96-well black-walled amine-coated tissue culture plate. Cells were incubated with FITC-Aβ_1–42_ (1 μM) at 37 °C 5 % CO_2_ for the times indicated in full growth medium. Cells were placed on ice and washed five times with ice-cold PBS^++^. One hundred microliters of PBS^++^ was added to each well, and FITC fluorescence was measured using a plate reader (excitation 494 nm, emission 521 nm).

Indirect immunofluorescence was used to determine subcellular localization of FITC-Aβ. IMG cells grown on glass coverslips were incubated for 1 h with FITC-Aβ and processed for fluorescence microscopy as described above. Briefly, cells were incubated with primary antibody targeting lysosomal-associated membrane protein 1 (LAMP1) (Pharmingen; 1:100 dilution). Secondary anti-rat rhodamine red antibody (JacksonImmuno Research; 1:1000 dilution) was used. Each antibody treatment was performed at room temperature for 1 h in 1 % BSA PBS^++^. Cells were then washed, mounted, and imaged as described above. Co-localized pixels were determined using ImageJ 1.48v software (National Institute of Health, USA).

### Statistical analysis

One-way ANOVA followed by Tukey’s multiple comparison test was used where indicated. Two-way ANOVA followed by Dunnett’s multiple comparison test was used where indicated. Paired *t* test statistical analysis was used where indicated. Statistical analyses were performed using Prism GraphPad version 6.00 for Windows, GraphPad Software, La Jolla, CA, USA.

## Results

### IMG cells display morphology similar to primary microglia and express the microglial markers CD11b and F4/80

**Fig. 1:**
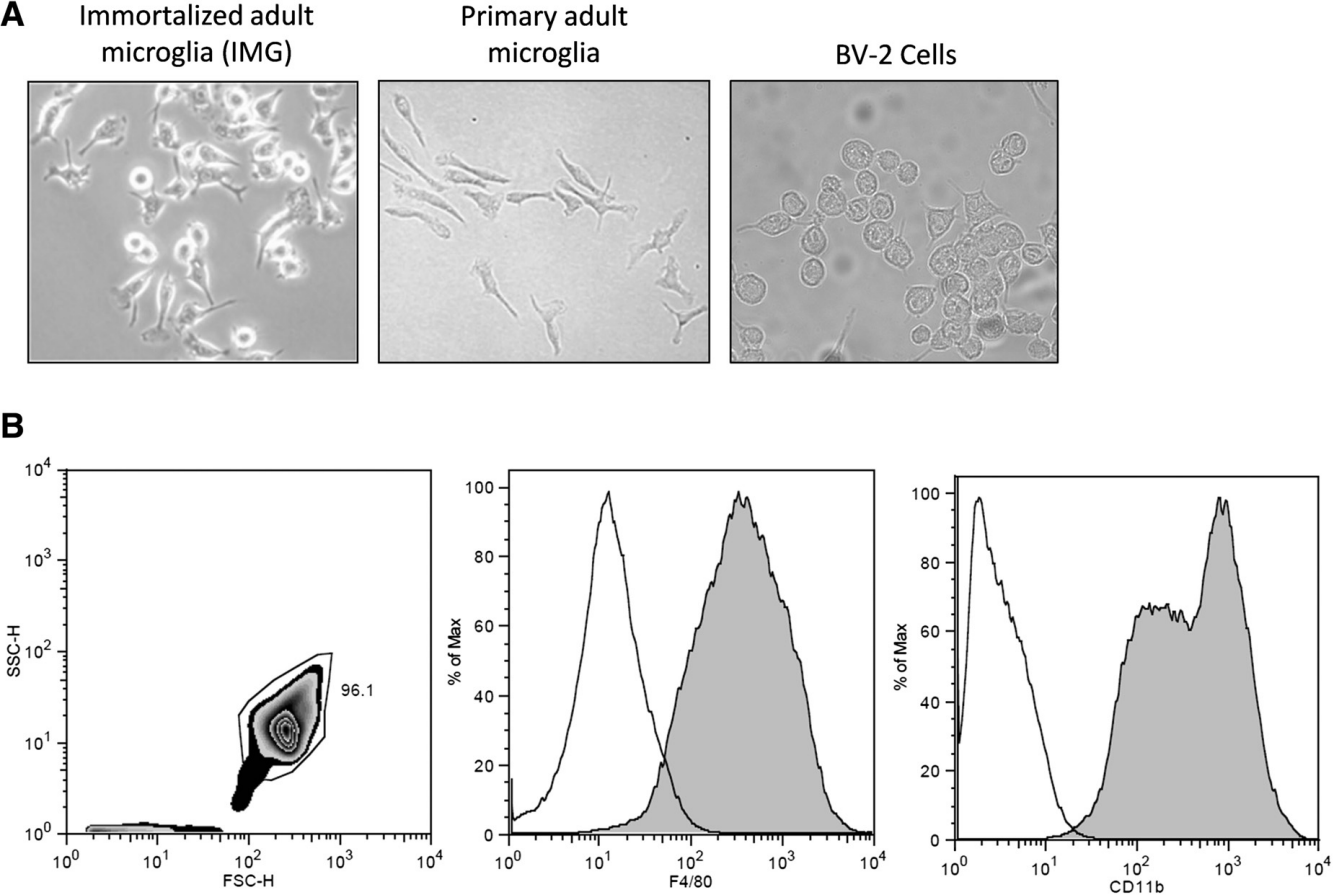
IMG cells display similar morphology to primary microglia and express the microglia markers CD11b and F4/80. **a** Representative DIC images of IMG, BV-2, and primary adult microglial cells. Images are at ×40 magnification. **b** Flow cytometry of IMG cells. Representative zebra plot (*left panel*) of IMG showing a population of >95 % was gated for the analysis of IMG F4/80 (*middle panel*) and CD11b (*right panel*) expression (*filled trace*) by flow cytometry. Isotype controls (*open trace*) are also included in the graphs

Phase-contrast images show that IMG, BV-2, and primary adult microglial cells are similar in cell morphology and size (Fig. 1a). The morphology of microglia is dependent upon their activation state; activated or dividing microglia are amoeboid-shaped whereas resting microglia display a ramified morphology [24]. Both IMG and BV-2 are rapidly dividing immortalized cells containing mostly amoeboid (dividing) with few ramified cells.

**Fig. 2:**
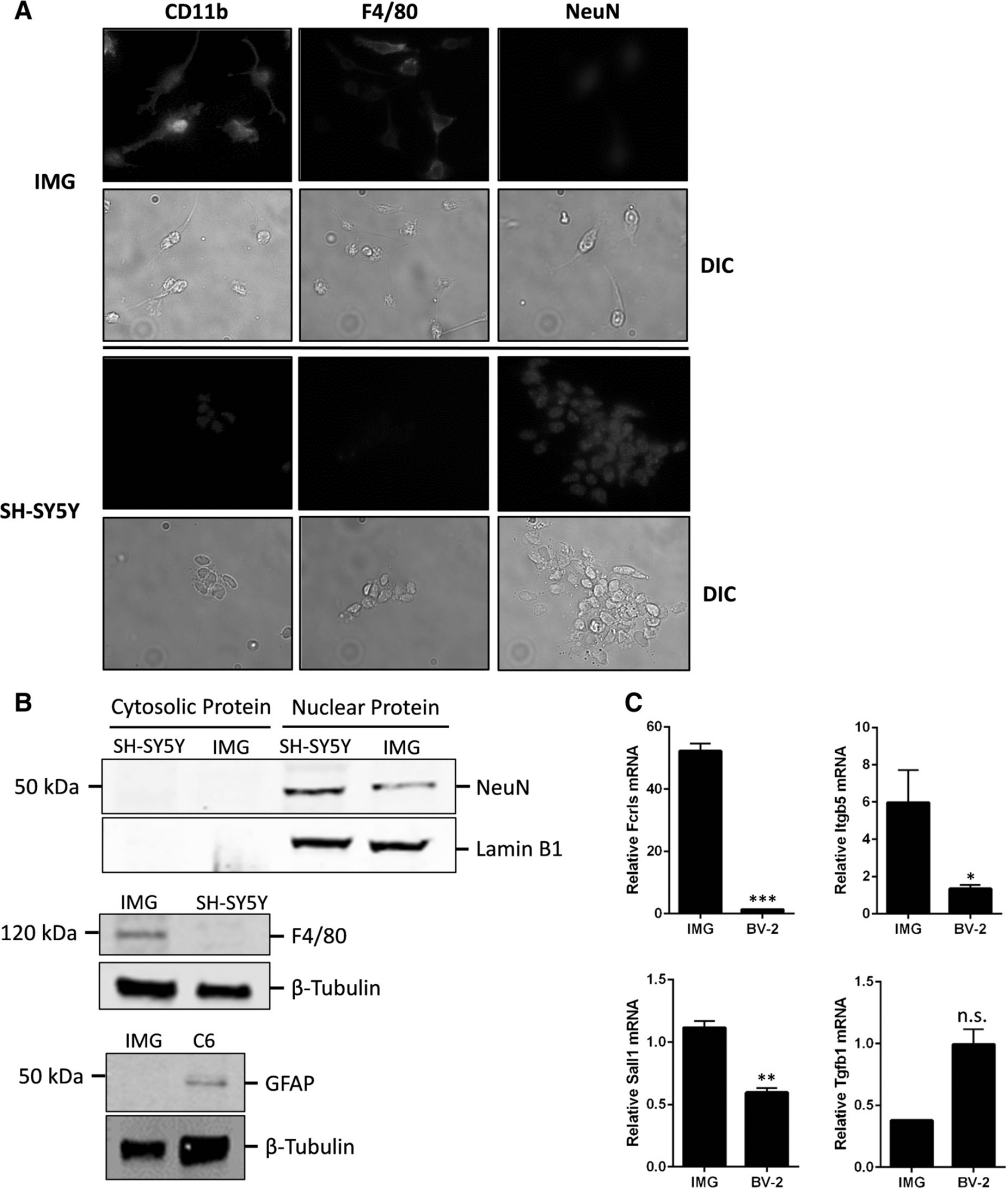
IMG cells express markers specific to microglia. **a** Immunofluorescence microscopy was used to assess the expression of the microglia markers CD11b and F4/80 by IMG and SH-SY5Y cells. Indirect immunofluorescence was used to assess the expression of the neuronal marker NeuN by IMG and SH-SY5Y cells. Images are at ×40 magnification. DIC images paired with each fluorescent image are shown. **b** Immunoblot of IMG and either SH-SY5Y or C6 glioma cell extracts. Whole-cell extracts are used unless otherwise indicated. Blots were probed for NeuN, GFAP, or F4/80. Loading controls were β-tubulin (whole cell) and Lamin B1 (nuclear extracts). **c** Quantitative PCR was used to assess transcript levels of the microglia-specific markers Fcrls, Itgb5, and Sall1 in IMG and BV-2 cells. A paired *t* test was used to determine the significance of the data. *P < 0.05; **P < 0.005; ***P < 0.001; *n.s.* is not significant. Data are represented as means ± s.d. (*n* = 3, technical replicates). Each experiment was repeated on at least two separate occasions

Expression of two microglia-specific markers, CD11b and F4/80, was determined by flow cytometry [3]. Both CD11b and F4/80 are detected in mouse brain after embryonic day 9.5 [16, 25]. Indeed, these markers are expressed by IMG cells as indicated by flow cytometry (Fig. 1b; filled trace); isotype controls are used to account for non-specific binding (Fig. 1b; open trace). We further confirmed the expression of CD11b and F4/80 using fluorescence microscopy (Fig. 2a); both CD11b and F4/80 were detected in IMG cells but not in SH-SY5Y cells, a neuronal cell line. Conversely, the neuron-specific marker NeuN was detected in SH-SY5Y cells and to a lesser extent in IMG cells (Fig. 2b). Western blot analysis confirmed NeuN staining was specific, as some protein was detected in IMG nuclear extracts. Western blot analysis also detected the astrocyte-specific marker GFAP in C6 glioma cell lysates but not in IMG cell lysates (Fig. 2b). Recent work has identified several genes unique to adult microglia [26]. Transcript levels of three of the four genes tested (Fcrls, Itgb5, Sall1, and Tgfb1) are significantly greater in IMG cells compared to BV-2 cells (Fig. 2c). These combined data suggest that IMG cells are a pure population with both morphology and biomarkers associated with adult microglia [16].

### IMG cells respond to exogenous pro- and anti-inflammatory stimuli

**Fig. 3:**
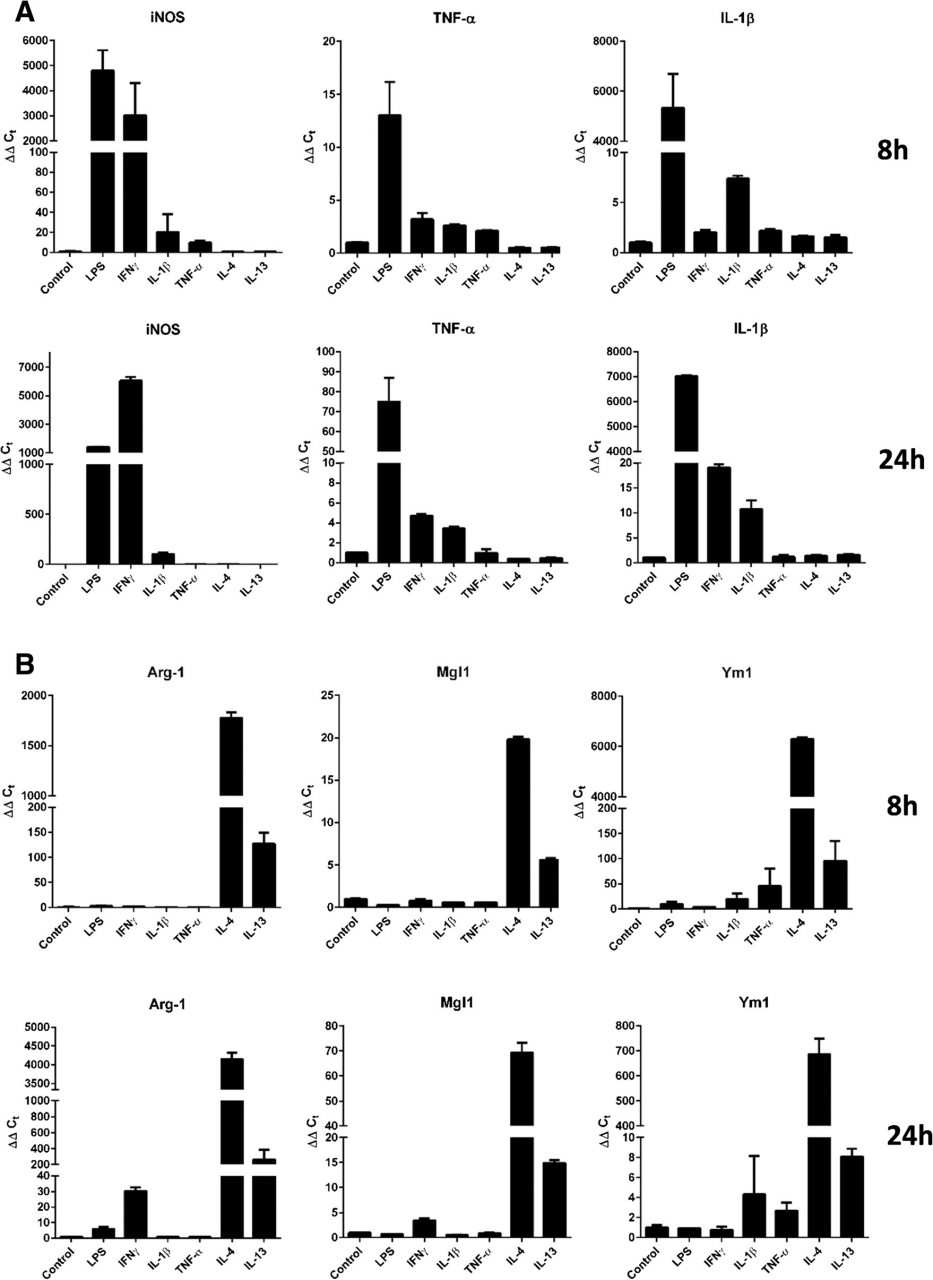
Response of IMG cells to pro- and anti-inflammatory stimuli. IMG cells were treated for either 8 or 24 h with LPS (10 ng/mL), interferon gamma (IFNγ) (10 ng/mL), IL-1β (10 ng/mL), TNF-α (5 ng/mL), IL-4 (10 ng/mL), IL-13 (10 ng/mL), IL-6 (10 ng/mL), or TGF-β (50 ng/mL). iNOS, TNF-α, and IL-1β message levels were used as markers for the microglial pro-inflammatory phenotype (**a**), whereas Arg-1, Mgl1, and Ym1 were used as markers for an anti-inflammatory phenotype (**b**). Quantitative PCR was used to assess the changes in message levels of the aforementioned genes relative to the internal control 36B4. Average of biological duplicates are shown with similar results obtained in at least two different experiments

Adult microglial cells characteristically polarize to either pro- or anti-inflammatory state in response to alterations in their local microenvironment [1]. A number of signaling molecules have been identified that induce reactive/reparative polarization of microglial cells. IMG cells were exposed for 8 or 24 h to induce either the reactive pro-inflammatory phenotype (LPS, IFNγ, IL-1β, and TNF-α) or the reparative anti-inflammatory phenotype (IL-4 and IL-13) [1, 3]. After incubation with the appropriate signaling molecules, RNA was isolated and qPCR was performed to determine the state of IMG activation. Markers indicative of reactive polarization that were assessed via qPCR included inducible nitric oxide synthase (iNOS), TNF-α, and IL-1β transcripts while markers indicative of the anti-inflammatory response included Arg-1, Mgl1, and Ym1 transcripts. The results show that IMG cells transition to a reactive phenotype when incubated for 8 or 24 h with LPS, IFNγ, and to a lesser extent IL-1β and TNF-α (Fig. 3a). Conversely, IMG cells express anti-inflammatory markers when incubated for 8 or 24 h with IL-4 and IL-13 (Fig. 3b). As similar changes in transcript abundance were noted in IMG cells treated with stimuli for 8 or 24 h, we performed all incubations with LPS or IL-4 for a period of 16 h in later experiments. Taken together, these data indicate that IMG cells retain the ability to polarize to the pro- or anti-inflammatory state, a functional feature of microglia that is well-documented by the literature [1].

**Fig. 4:**
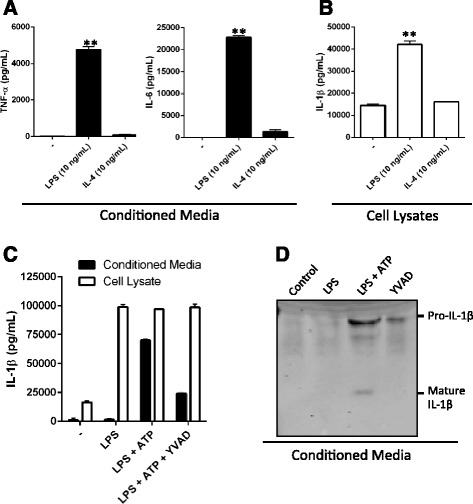
IMG cells respond to LPS treatment by increasing protein expression of inflammatory cytokines. **a** ELISA was used to analyze the secretion of TNF-α and IL-6 into IMG cell-conditioned medium during a 16-h incubation with or without LPS (10 ng/mL) or IL-4 (10 ng/mL) as described in the “Methods” section. **b** ELISA analysis of IL-1β in whole-cell lysates of IMG cells treated as described in (**a**). **c** ELISA of conditioned media (*filled bars*) and cell lysates (*open bars*) collected from IMG cells treated with or without LPS (10 ng/mL) for 6 h. LPS-treated cells were also incubated with or without Ac-YVAD-CMK (40 μM; YVAD) for 5 min, followed by ATP (5 mM) for 30 min as indicated. **d** Immunoblot analysis of conditioned media from IMG cells treated as described in (**c**). Blots were probed for IL-1β. Pro- (34 kDa) and mature (17 kDa) IL-1β bands are indicated. One-way ANOVA was used to determine the significance of the data. *P < 0.01; **P < 0.0001. Data are represented as means ± s.d. (*n* = 3, technical replicates). Each experiment was repeated on at least two separate occasions

As IMG cells are activated by incubation with LPS (Fig. 3a), we hypothesized protein production of secreted pro-inflammatory factors would increase. To test this, an ELISA was used to determine relative abundance of the cytokines IL-6 and TNF-α in IMG cell-conditioned media after incubation with LPS. ELISA results confirmed that exposure to LPS for 16 h yielded a significant increase in IL-6 and TNF-α (Fig. 4a); no significant change in expression of these cytokines was noted when cells were exposed to IL-4 for 16 h. LPS also increased synthesis of IL-1β, detected by ELISA in IMG cell lysates (Fig. 4b). Inflammasome activation and caspase-1-mediated cleavage of pro-IL-1β is required for secretion of mature IL-1β by LPS-primed microglia [27]. Activation of the NLRP3 inflammasome in LPS-primed IMG cells by treatment with ATP significantly enhanced secretion of IL-1β into conditioned media (Fig. 4c); addition of the caspase-1 inhibitor Ac-YVAD-CMK abrogated this effect. Treatment of LPS-primed IMG cells with ATP resulted in processing and secretion of the 17-kDa mature form of IL-1β into the media (Fig. 4d). Cleavage to the mature form was inhibited by Ac-YVAD-CMK (Fig. 4d). Thus, LPS upregulates synthesis, processing, and secretion of mature IL-1β by IMG cells in an appropriate two-step process.

### IMG cells display a more robust response than BV-2 cells to pro- and anti-inflammatory stimuli

**Fig. 5:**
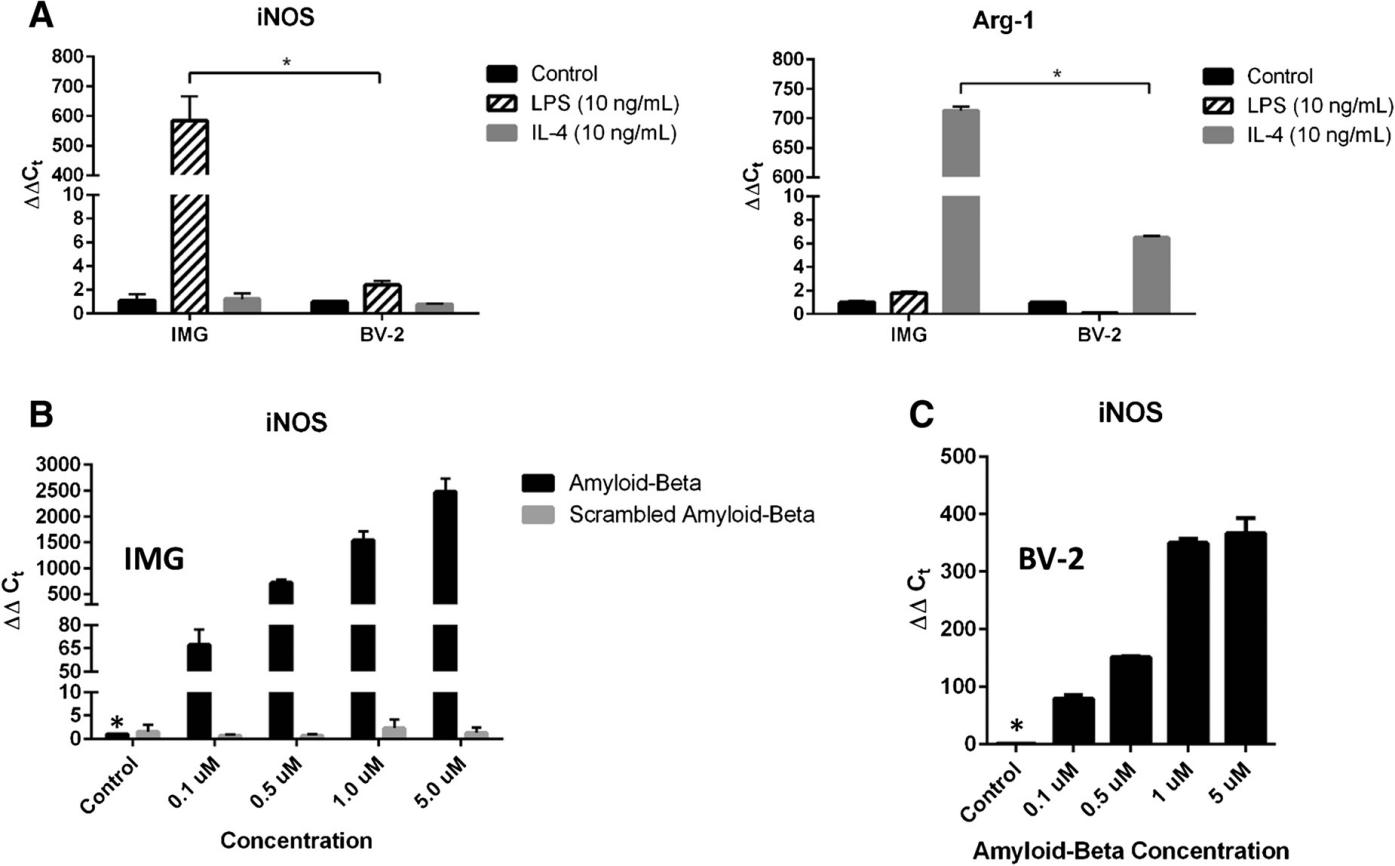
IMG cells are significantly more sensitive to polarization by LPS, Aβ, and IL-4 than are BV-2 cells. **a** Quantitative PCR was used to analyze the transcript abundance of iNOS and Arg-1 in IMG cells and BV-2 cells treated with or without LPS (10 ng/mL) or IL-4 (10 ng/mL) for 16 h. **b**, **c** IMG and BV-2 cells were treated with Aβ_1–42_ peptide or scrambled Aβ_1–42_ peptide at varying concentrations as indicated for 6 h. The transcript abundance of iNOS was determined via qPCR. One-way (**b**) and two-way (**a**) ANOVA statistical analyses were used to determine the significance of the data. *P < 0.0001. Data are represented as means ± s.d. (*n* = 3, technical replicates). Each experiment was repeated in triplicate

BV-2 cells have been often used to examine microglial cell function. However, this cell line responds poorly to pro- and anti-inflammatory stimuli (LPS and IL-4, respectively) [14, 28–31]. We directly compared IMG and BV-2 responses to LPS or IL-4 upon 16-h exposure. Transcript abundance of both iNOS and Arg-1 was monitored via qPCR (Fig. 5). IMG cells increase the production of iNOS (>200-fold) or Arg-1 (>100-fold) transcripts compared to BV-2 cells upon exposure to either LPS or IL-4, respectively.

In the Alzheimer’s brain, microglia are polarized to a reactive state by Aβ peptides in the interstitium. We compared the efficacy of Aβ on iNOS production in IMG and BV-2 cells. The transcript abundance of iNOS within both IMG and BV-2 cells increased as a consequence of increasing Aβ concentration (Fig. 5b, c); scrambled Aβ peptide had no measurable effect. Notably, the increase in Aβ-induced iNOS expression by IMG cells (approx. 2500-fold at 5 μM Aβ) was far greater than that observed in BV-2 cells (approx. 350-fold at 5 μM Aβ). IMG cell nitrite production was increased with Aβ treatment as determined by the Griess assay (data not shown).

### IMG cells phagocytose foreign particles

**Fig. 6:**
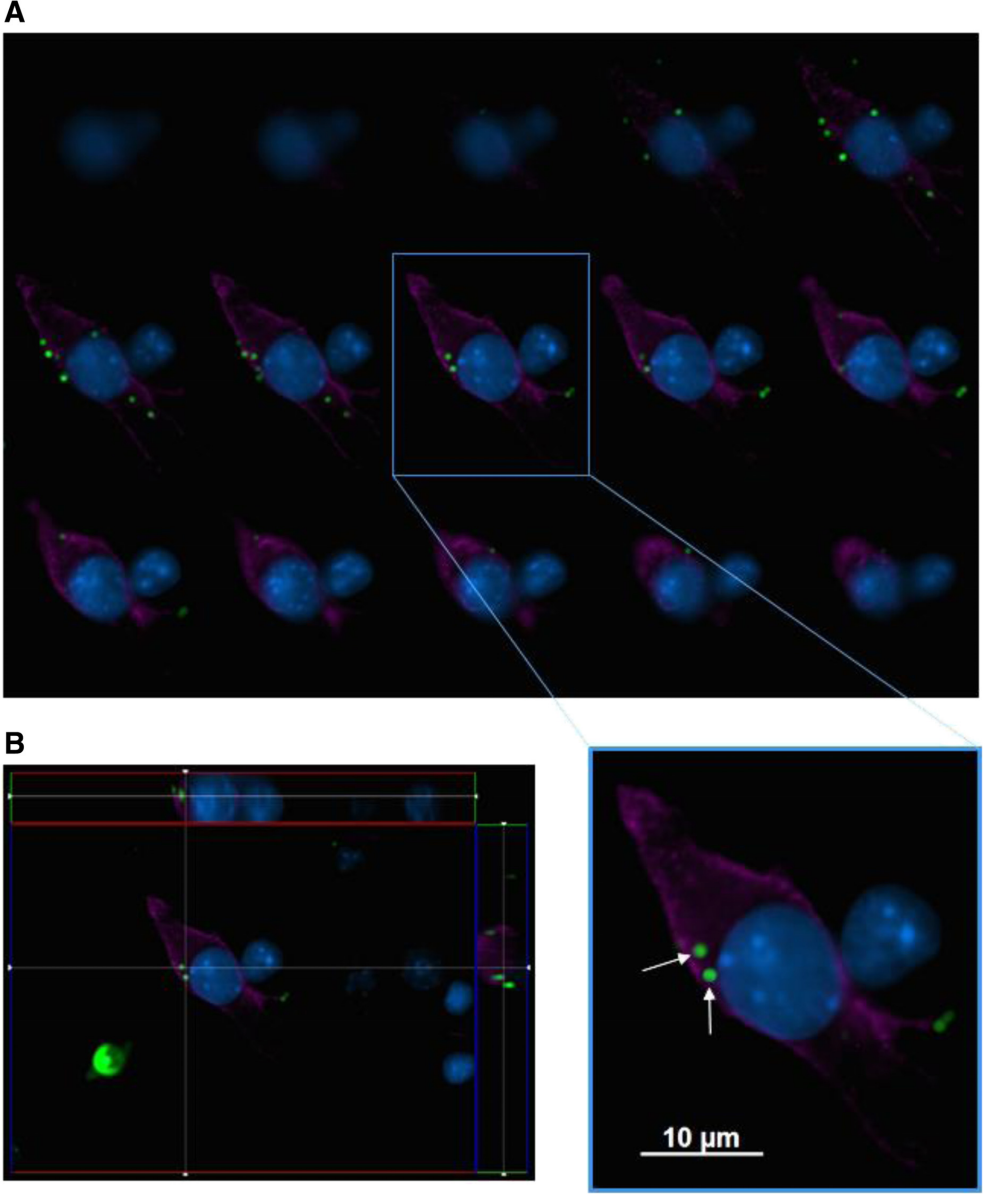
IMG cells internalize fluorescent latex beads. Images of IMG cells that were incubated with fluorescent latex beads for 1 h at 37 °C 5 % CO_2_ were obtained. CD11b (*purple*) was used as a marker for IMG cells. **a** Z-stack series of microscopic images of an IMG cell (*purple*) were compiled into a grid to demonstrate the internal localization of two fluorescent latex beads (*green dots*; indicated by *arrows*). **b** Z-stack series from (**a**) is accompanied by two cross-sectional perspectives. The images were obtained with a ×100 objective. DAPI (*blue*) stains the nucleus

When confronted with foreign particles such as dead or dying neurons, microglia will remove these materials via phagocytosis [11, 32]. To test their phagocytic potential, IMG cells were exposed to 1-μm-diameter carboxylate-modified polystyrene fluorescent YG beads for 1 h at 37 °C. Internalization of the YG beads by IMG cells was evaluated by fluorescence microscopy. A series of z-stack images were acquired to examine the localization of YG beads (shown in green) within a single IMG cell (identified by the microglial marker CD11b (purple)) (Fig. 6a). The compilation of images provided by the z-stack clearly indicates that this particular IMG cell engulfed at least two YG beads, as shown by their intracellular localization (Fig. 6a, arrows). Cross-sectional images of the same IMG cell are shown in Fig. 6b.

**Fig. 7:**
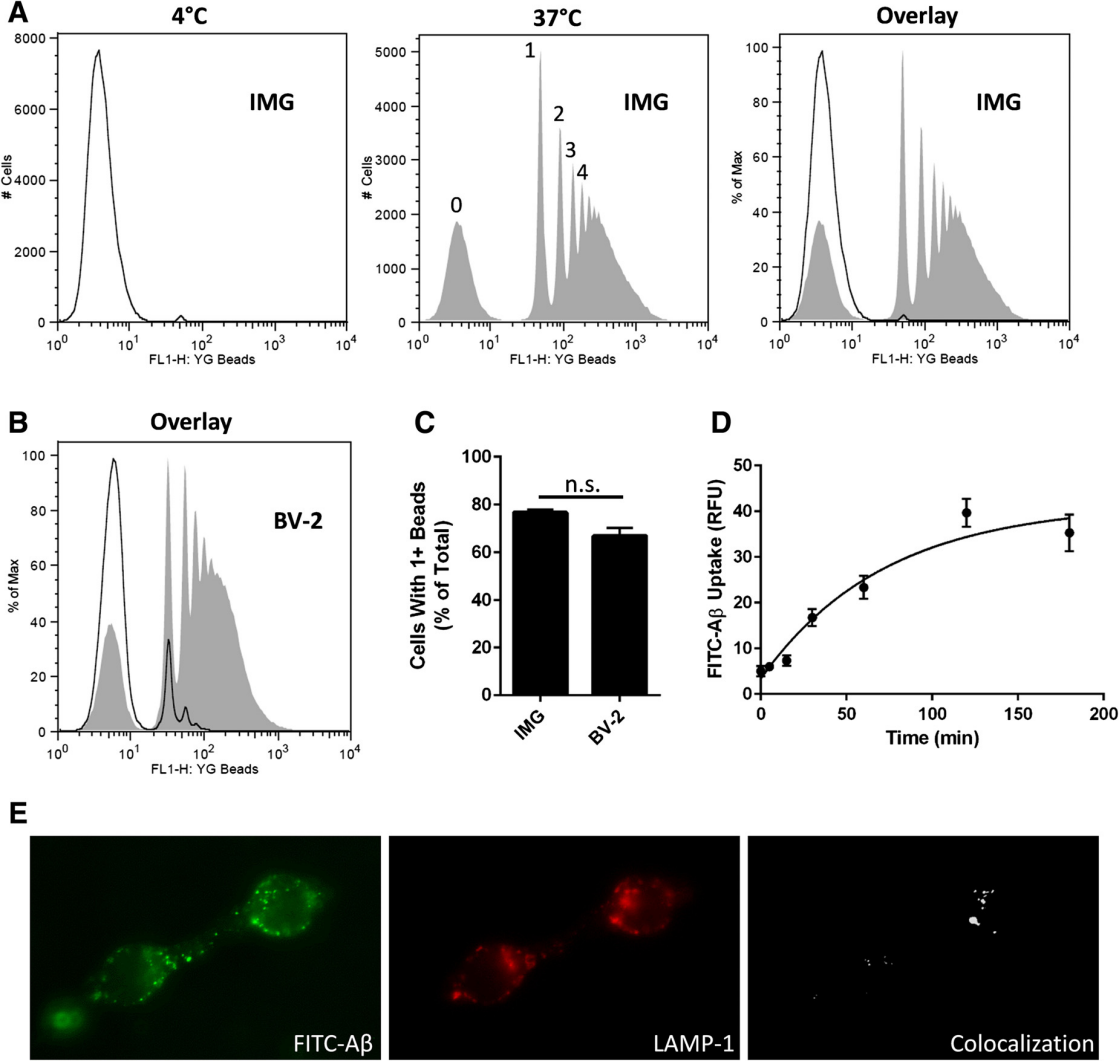
IMG cells retain phagocytic properties inherent in microglial cells. Phagocytosis of 1-μm fluorescent yellow-green latex beads (YG beads) by IMG (**a**) or BV-2 (**b**) cells as analyzed by flow cytometry. Cells were incubated with the YG beads for 1 h at either 4 °C (*open trace*) or 37 °C (*gray fill*) prior to analysis by flow cytometry. Each *peak* represents the IMG population which has ingested either 0, 1, 2, 3, or 4 beads, respectively. Each *graph* (**a**, **b**) is a representation of data from at least three biological replicates. **c** Percentage of cells which acquired ≥1 YG beads as analyzed by flow cytometry. **d** Kinetic analysis of oligomeric FITC-Aβ uptake by IMG. IMG cells were incubated with FITC-Aβ (1 μM) for the indicated times at 37 °C. IMG-associated FITC fluorescence was measured using a plate reader. **e** Subcellular localization of internalized FITC-Aβ was assessed using fluorescence microscopy. IMG cells were incubated for 1 h with FITC-Aβ (*green*) prior to being processed for microscopy. Phagolysosomes were identified using LAMP1 antibody (*red*). Areas of co-localization (*white*) were determined using ImageJ software. A paired *t* test was used to determine the significance of the data (*n.s.* is not significant). Data are represented as means ± s.d. (*n* = 3, experimental replicates)

Next, we used flow cytometry to quantify the number of beads phagocytosed per IMG cell. Again, IMG cells were incubated with YG beads for 1 h at 37 °C to allow time for phagocytosis to occur. Simultaneously, a dish of IMG cells was incubated with YG beads for 1 h at 4 °C to account for non-specific binding. After exposure to YG beads, IMG cells were washed thoroughly and analyzed for yellow-green fluorescence via flow cytometry. By monitoring the degree of fluorescent intensity, populations of IMG cells which have ingested 0, 1, 2, 3, or 4 beads can be differentiated (Fig. 7a, see labels in the middle graph). These data are quantitatively similar to phagocytosis of YG beads by BV-2 cells (Fig. 7b, c).

In the Alzheimer’s brain, a major function of microglial is to clear Aβ by phagocytosis. To study uptake of Aβ, IMG cells were incubated at 37 °C with 1 μM FITC-Aβ for the indicated times and washed, and then internalization was analyzed using a fluorescence plate reader. These data show that IMG phagocytosis of FITC-Aβ is time-dependent and maximal within 3 h (Fig. 7d). We hypothesized that a portion of the Aβ phagocytosed by IMG cells would be trafficked to the phagolysosome within this time frame. Immunofluorescence microscopy confirmed that a fraction of the FITC-Aβ phagocytosed by IMG during a 1-h incubation period co-localized with the LAMP1 (Fig. 7e).

### Aβ-induced iNOS expression by IMG cells is blocked by cannabinoids

**Fig. 8:**
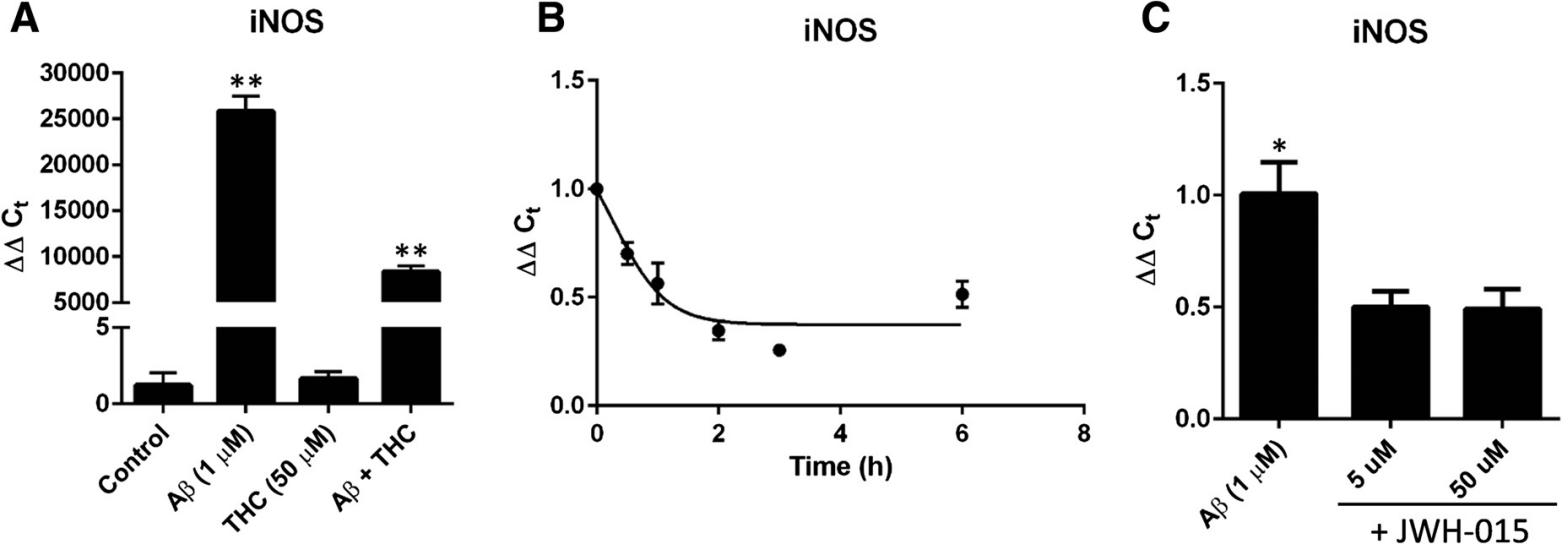
Cannabinoids inhibit Aβ-induced iNOS production in IMG cells. Quantitative PCR was used to analyze the transcript abundance of iNOS in IMG cells. **a** IMG cells were treated with or without Aβ and/or THC for 6 h at 37 °C. IMG cells were incubated with Aβ (1 μM; 6 h) and the CB_2_ selective agonist JWH-015 (50 μM) for the indicated times (**b**) or at varying concentrations (**c**). One-way ANOVA statistical analyses were used to determine the significance of the data. *P < 0.005; **P < 0.0001. Data are represented as means ± s.d. (*n* = 3, technical replicates)

Since IMG cells display the same characteristics as primary microglia, the idea that this cell model system could be used for candidate drug screens in the treatment of Alzheimer’s disease is well-supported. To evaluate their use in pharmacological studies, we examined the effects of the cannabinoids THC and JWH-015 on Aβ-induced iNOS gene expression. Prolonged use of cannabinoids is reported to reduce inflammation in the brains of Alzheimer’s disease model mice [33]; cannabinoids have also been shown to reduce microglial activation both in vitro and in vivo [34, 35]. The principal psychoactive constituent of cannabis, THC, significantly inhibited the Aβ-induced increase in iNOS transcript levels within IMG cells (Fig. 8a). THC is an agonist to both cannabinoid receptor type 1 (CB_1_; psychoactive) and type 2 (CB_2_; non-psychoactive). JWH-015 is a selective CB_2_ agonist. Like THC, JWH-015 (50 μM) inhibited Aβ-induced iNOS expression and in a time-dependent manner (Fig. 8b). In a dosing experiment, we determined the effect of JWH-015 on Aβ-induced IMG iNOS levels is reproducible even at a lower dose (5 μM) (Fig. 8c). Neither drug altered the expression of the M2 marker Arg-1 in Aβ-treated IMG cells (data not shown). These data are consistent with previous results in the literature demonstrating cannabinoid-mediated prevention of Aβ-induced microglial activation [35]. Combined, this evidence demonstrates the pharmacological intervention of Aβ-induced M1 activation of IMG cells.

## Discussion

Chronically activated pro-inflammatory adult microglial cells contribute to the progression of neurodegenerative diseases like Alzheimer’s and Parkinson’s diseases [4, 5, 9, 10]. An adult immortalized microglial cell model system for examination of neuroinflammation is therefore essential. BV-2 cells, a currently used model system, were generated using mononuclear cells from an embryonic mouse brain. A major drawback is the limited BV-2 cell response to pro- and anti-inflammatory stimuli (Fig. 6) [14, 15, 29–31]. On the other hand, primary adult microglial cell isolation yields a limited number of cells, can be technically challenging with potential cell activation, and suffers from a lack of homogeneity.

Here, we describe the development of a novel immortalized adult microglial cell line, IMG cells, which share phenotypic attributes with primary adult microglia and respond robustly to inflammatory signals. Primary adult microglia react to changes in their extracellular environment by polarizing to a reactive phenotype or one that promotes resolution of inflammation [10, 36]. IMG cells are far more responsive than BV-2 cells to external pro- and anti-inflammatory signals such as LPS and IL-4. The fact that IMG cells display a robust response to external pro- and anti-inflammatory stimuli make this an ideal model system to examine microglial involvement in neurodegenerative diseases, such as Alzheimer’s disease, where a link between chronic microglial activation and the progression of the disease has been well established [5, 37].

In the early stages of Alzheimer’s disease, brain microglia become activated by Aβ peptides which, in turn, are cleared by the microglia via phagocytosis [11, 38–40]. The activation of microglia by Aβ peptides increases secretion of pro-inflammatory cytokines, which can reduce microglial cell Aβ peptide receptor expression via an autocrine feedback loop [38, 41]. The reduced expression of Aβ receptors by microglia results in reduced clearance, thus promoting plaque formation to persist and disease progression to continue. Secretion of pro-inflammatory cytokines by activated microglia also promotes neuronal cell damage and death. It is therefore important to understand microglial cell function, especially Aβ-induced activation and phagocytosis, in order to develop new therapies aimed at targeting these pro-inflammatory effects. We have developed and characterized the IMG cell line to advance towards these goals.

Taming the chronic pro-inflammatory polarization of microglia in Alzheimer’s disease is one therapeutic strategy that is actively investigated [2]. In the past, BV-2 cells have been used to screen for potential anti-inflammatory drugs to quell or negate the effect of LPS [30]. Our experiments show that the IMG cells’ response to inflammatory factors is far greater than that of BV-2 cells, raising the promise of their utility in such screening efforts. Moreover, IMG cells are highly reactive to Aβ, establishing an in vitro model relevant to Alzheimer’s disease. To explore their potential use in pharmacological studies, we established that the cannabinoids THC and JWH-015 limit Aβ-induced IMG cell inflammation. The mechanism of anti-inflammatory action of cannabinoids has yet to be established, but our studies suggest CB2 activation by the selective agonist JWH-015 is sufficient to mediate this effect. In preliminary experiments, we have determined that cannabinoids do not alter Aβ uptake or degradation by IMG cells (data not shown). CB2 may elicit downstream signals to reduce the Aβ-induced inflammatory response in IMG cells. Potential targets include elements of the peroxisome proliferator-activated receptor-γ (PPAR-γ) pathway [42]. Further investigation is required to determine the exact mechanism of cannabinoid drug action on IMG cells and to employ these cells to screen for new drug candidates that ameliorate Aβ-induced inflammation.

## Conclusions

We have established an immortalized cell line from adult murine microglia. We conclude this model system, called IMG for immortalized microglia, fully recapitulates morphological and functional characteristics of brain microglia. IMG cells express microglia-specific markers and respond appropriately to pro- and anti-inflammatory stimuli. IMG cells phagocytose foreign particles. Moreover, IMG cells are robustly activated by Aβ_1–42_, which they also phagocytose. The response of IMG cells to extracellular pro- and anti-inflammatory stimuli, including Aβ, is far greater than the most commonly employed microglial BV-2 cell line. As an example of their potential utility, we demonstrate administration of cannabinoids can effectively alleviate Aβ activation of IMG cells. This cell line provides a new platform to explore drug interactions and gain mechanistic information about neuroinflammatory responses underlying Alzheimer’s disease and other neurological disorders.

## Additional file

Additional file 1: Figure S1. Dot blot analyses using species-specific antibodies. Preparation of Aβ contains both oligomeric and fibrillar Aβ. Immunoreactivity of dot blots of Aβ scrambled (Aβscr) or Aβ1-42 was detected using 4G8, A11, or OC antibodies as described in the “Methods” section. Scrambled Aβ demonstrates antibody specificity. (PDF 155 KB)
