# Supplementary material for: Characterization of a novel adult murine immortalized microglial cell line and its activation by amyloid-beta
Source: J Neuroinflammation. 2016 Jan 27;13:21. doi: 10.1186/s12974-016-0484-z (PMC4730646; doi:10.1186/s12974-016-0484-z)
Supplement: Additional file 1: Figure S1. — Dot blot analyses using species-specific antibodies. Preparation of Aβ contains both oligomeric and fibrillar Aβ. Immunoreactivity of dot blots of Aβ scrambled (Aβscr) or Aβ1-42 was detected using 4G8, A11, or OC antibodies as described in the “Methods” section. Scrambled Aβ demonstrates antibody specificity. (PDF 155 KB) [file 12974_2016_484_MOESM1_ESM.pdf]

Figure S1 Preparation of Aβ contains both oligomeric and fibrillar Aβ. Immunoreactivity of dot blots of Aβ scrambled (Aβscr) or Aβ1-42 was detected using 4G8, A11, or OC antibodies as described in methods. Scrambled Aβ demonstrates antibody specificity.

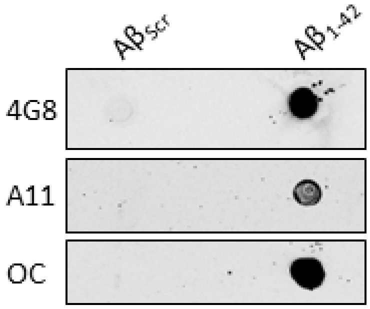

Supplementary materials and methods

The $A\beta_{1\text{-}42}$ peptide was prepared as described in the materials and methods section. Both the scrambled Aβ and $A\beta_{1\text{-}42}$ were dotted onto a nitrocellulose membrane and allowed to dry. The membranes were blocked for 1h with TBST plus 10% milk at room temperature. After three 5 minute washes with TBST, the membranes were incubated with either primary mouse anti-Aβ (4G8) antibody (1:1000 dilution; BioLegend), rabbit anti-Aβ oligomers (A11) antibody (1:1000 dilution; Rockland, Inc.), or rabbit anti-Aβ fibrils (OC) antibody (1:1000 dilution; Rockland, Inc.) in TBST plus 5% milk over the weekend at 4°C. After three 10 minute washes with TBST, the membranes were incubated for 1h at room temperature with either IRDye 800CW donkey anti-mouse or anti rabbit IgG (1:5000 dilution; Li-Cor, Lincoln, NE) in TBST 5% milk. The membranes were washed three times for 5 minutes and then imaged using Li-Cor Odyssey 2.1 infrared detection technology.
